# Associations between Metal Exposures and Cognitive Function in American Older Adults

**DOI:** 10.3390/ijerph19042327

**Published:** 2022-02-17

**Authors:** Nozomi Sasaki, David O. Carpenter

**Affiliations:** 1 Department of Environmental Health Science, School of Public Health, University at Albany, Rensselaer, NY 12144, USA; 2 Institute for Health and the Environment, University at Albany, Rensselaer, NY 12144, USA

**Keywords:** cognitive impairment, cognitive functions, metal exposures, aging, NHANES, CERAD, DSST, selenium, lead, cadmium

## Abstract

Cognitive function frequently declines with older age, independently of the development of neurodegenerative diseases, and few interventions are known to counter this decline. Exposure to neurotoxic metals may contribute to this decline in cognitive function in older adults. Using the National Health and Nutrition Examination Survey (NHANES) data, the performance of 3042 adults aged 60 years and older on three cognitive tests for immediate, delayed, and working memory were examined in relation to blood concentrations of seven metals and metalloids and urinary concentrations of nineteen metals and metabolites. Using linear regression models, associations between cognitive tests and logarithms of metal exposures were adjusted for age, sex, ethnicity, education level, depression, diabetes, alcohol consumption, and cigarette use. Increased selenium was strongly associated with better performance on all three cognitive tests. Cadmium and lead were negatively associated with performance on all three cognitive tests. Some urinary metabolites of arsenic, urinary lead, cadmium, and tungsten were significantly associated with poor performance on some tests. In older adults, higher selenium levels were strongly associated with better cognitive performance.

## 1. Introduction

Cognitive function often declines with age, even in people without neurodegenerative diseases, although the rate of decline shows significant heterogeneity among individuals. In spite of television ads promoting pills to improve cognitive function in older people, there is a lack of evidence as to how to avoid cognitive decline, and even a lack of knowledge of the direct cause of cognitive decline with age. Because several metals are known to reduce learning and memory, we explored NHANES data to examine whether metal exposures were important factors influencing cognitive function in older adults.

Exposures to lead [1,2,3], cadmium [4], methylmercury [5,6], and arsenic [7,8,9] are known to reduce cognitive function and cause neurobehavioral effects in children. Lead exposures have been linked to declining child neurodevelopment, verbal abilities, and attention [1]. Lead concentrations as low of 5 μg/dL had significant adverse effects on attention, arithmetic, and reasoning scores among children aged 6–16 years in a cross-sectional study [2]. Furthermore, from a longitudinal study, it was found that the rate of decline in IQ at 3 and 5 years of age was steeper at lower than at higher concentrations of postnatal lead exposures [3]. While prenatal cadmium exposures have not been shown to be significantly associated with children’s cognitive performance at ages 1–8 years in prospective pregnancy and birth cohorts, negative effects on cognitive function are suggested in animal models [4,10]. Prenatal methylmercury exposures result in declining language, attention, and memory abilities at age 7 years, and declining IQ was observed at age 22 years [5,6]. Arsenic exposures in children aged 6–8 years have been linked to reduced cognitive performance even after adjusting for the effects of lead [7,8]. School-aged children with prenatal arsenic exposures showed a lasting negative effect on cognitive performance relating to full developmental scores, verbal comprehension, perceptual reasoning, and processing speed [9].

Compared to research in children, there has been less research on the effects of exposure to metals on cognitive function in older adults. However, available information shows that these metals are associated with lower adult cognition. A decline in cognitive function often has a devastating effect on the quality of life in older adults, creating fear of developing neurodegenerative diseases. From longitudinal cohort studies, higher lead concentrations in the tibial bone were associated with lower performance on language, motor ability, verbal memory and learning, and visual memory tests in adults aged 50–70 years [11,12]. In older men, higher lead in the patella (knee-cap) bone from non-occupational exposure was associated with a steeper cognitive decline over an average 3.5 year period in a longitudinal study [13]. Workers exposed to lead in young adulthood showed progressive cognitive decline over two decades [14]. In a prospective study, cumulative low-level exposures to lead (mean ± SD was 10.5 ± 9.7 μg/g in the tibia bone) were associated with poorer cognitive performance in women aged 47–74 years [15]. From a longitudinal study, a higher lead concentration in the patella bone (IQR = 21 µg/g) was associated with faster cognitive decline in the language and memory domains [16]. These studies suggest that lead accumulation in the body burden may contribute to cognitive decrement with aging.

A cross-sectional study among U.S. adults aged 20–59 years found that cadmium exposure from the diet was responsible for reduced cognitive performance in attention and perception from NHANES data [17]. Both current and long-term arsenic exposures from groundwater in rural-dwelling adults aged 40–96 years were significantly associated with poor performance on language, visuospatial skills, executive functioning, and immediate and delayed memory in cross-sectional studies [18,19]. Methylmercury exposures from fish consumption in adults aged 17 years and older have been negatively associated with performance on fine motor speed and dexterity, concentration, and verbal learning and memory tests in a cross-sectional study [20]. These results suggest that neurotoxic metals reduce cognitive function not only in children, but also in adults.

There is some evidence that higher plasma concentrations of selenium are associated with lower rates of cognitive decline among older adults, which suggests that elevated concentrations of selenium might be protective [21]. Selenium is an essential nutrient, but one that is toxic at high concentrations [22]. There are at least 25 selenium-containing proteins in humans [23]. At least nine of these are present in the brain, regulating cellular functions, such as redox signaling and calcium regulation, and serving as antioxidant enzymes [24,25]. One group of particularly important selenium-containing enzymes related to cognitive function are the type 2 and 3 deiodinases that convert thyroxine (T4) to triiodothyronine (T3) [26]. While T4 is the primary hormone released by the thyroid gland, the active form is T3, and inadequate production of T3 can cause hypothyroidism. Low ratios of T3/T4, common in the elderly, are usually secondary to selenium deficiency [27]. Subclinical or overt hypothyroidism is common with aging and is often associated with cognitive decline [28].

Overall, findings on metal exposures and cognitive function suggest negative and/or positive associations in older adults. In the present study, we examined cross-sectional associations to understand which metals or metabolites have associations with cognitive function tests relating to immediate learning and recall, delayed recall, and working memory in older adults using data from the NHANES. We have examined the associations of all metals, metalloids, or metabolites on cognitive function for which biomonitoring information is available from whole blood or urine in the NHANES from 2011 to 2014. This includes cadmium, lead, manganese, selenium, inorganic mercury, methylmercury, and total mercury in whole blood, and urinary arsenous acid, arsenobetaine, arsenocholine, dimethylarsinic acid, monomethylarsonic acid, total arsenic, barium, cadmium, cesium, cobalt, lead, manganese, molybdenum, strontium, thallium, tin, tungsten, and uranium.

## 2. Materials and Methods

### 2.1. Study Population

We obtained data from the 2011–2014 NHANES, a national survey assessing health and nutritional status administered to a multistage probability sample of the general U.S. population conducted by the National Center for Health Statistics (NCHS) of the Centers for Disease Control and Prevention (CDC) [29,30]. Participants completed surveys about demographics, health history, and diet, and they submitted blood and urine samples during physical examinations. Concentrations of metals and metabolites in the blood and urine were measured in a subsample following certified laboratory methods using sensitive instruments. In the NHANES 2011–2014, participants were randomly assigned into subsample groups A, B, and C and, based on the subsample group, the biomonitoring of environmental chemicals was performed [29]. The subsampling was necessary because one cannot obtain adequate amounts of blood in order to monitor all chemicals of interest, so different chemicals were measured among the different subgroups. The study protocol has been described previously [29]. The wordlist and learning module for immediate and delayed memory from the Consortium to Establish a Registry for Alzheimer’s Disease (CERAD) and for working memory from the Digit Symbol Substitution Test (DSST) were administered to adults aged 60 years and older who passed a cognitive function screening test [29]. The top-coded age was 80 years in the NHANES due to participants’ confidentiality. Hence, our study population included adults aged 60–80 years who passed cognitive screening tests and for whom metals and/or metabolites were measured in whole blood and urine samples.

### 2.2. CERAD Modules

Tests for immediate learning and recall and delayed recall were assessed by CERAD modules [29]. The tests consist of three consecutive learning and immediate recall trials and one delayed recall assessment. They were administrated by trained interviewers, and answers were audio-recorded for quality control. Participants chose their preferred language from English, Spanish, Korean, Vietnamese, and Chinese. CERAD scores were confirmed by a second consultant using the audio recordings. For the learning and recall assessments, three learning trials were conducted. Participants were asked to read aloud 10 unrelated words, displayed on a monitor, one at a time, and then to immediately recall as many as possible. The order of the 10 words was changed in each learning trial. The total immediate learning and recall (CERAD immediate recall) scores were calculated by summing the three test scores for each participant (range: 0 to 30). To assess delayed recall capability, 10 min after the first learning trial’s completion, the participants were asked to recall as many words as possible. The delayed recall score was the sum of words recalled (range: 1 to 10).

### 2.3. The DSST Module

This module from the Wechsler Adult Intelligence Scale tests processing speed, sustained attention, and working memory [29,31]. The test consists of a series of nine numbers paired with symbols. After an initial screening by practice pre-tests, eligible participants are given only two minutes to copy the appropriate symbols with numbers on a 133-box grid. The score is the total number of boxes with the correct markings (range: 0 to 133).

### 2.4. Analysis of Metal Concentrations

Exposures to metals were determined by analyses of whole blood and/or urine samples from randomly selected participants in each NHANES cycle. Collected samples were stored at a temperature of −70 °C and shipped to the National Center for Environmental Health. Certified laboratory methods, using sensitive instruments with careful quality assurance and quality control, were utilized [29]. All concentrations of metals and metabolites were measured with inductively coupled plasma-mass spectrometry (ICP-MS) [29]. The protocols of quality assurance and control were performed to meet the 1988 Clinical Laboratory Improvement Amendments mandates [29]. The limits of detection (LOD) and participants numbers with value below the LOD are listed in Table S2.

### 2.5. Statistical Analyses

Our statistical analyses had 2 stages: first, single metal or metabolite analyses, and second, multiple metals or metabolites from the stage 1 results. The distributions of CERAD and DSST scores by each metal were determined. For each analysis, only participants who had recorded metal concentrations above the LOD were included. This process improved the variance of metal concentrations with log-normal distributions. Concentrations of lead, cadmium, and total mercury in the whole blood and urinary cadmium, cesium, molybdenum, manganese, lead, and tin were log-transformed. Additionally, concentrations of selenium, inorganic mercury, urinary antimony, strontium, thallium, tungsten, and uranium were truncated to the 95th percentile values and log-transformed. Concentrations of manganese and methylmercury and urinary concentrations of total arsenic, arsenous acid, arsenobetaine, arsenocholine, dimethylarsinic acid (DMA), monomethylarsonic acid (MMA), barium, and cobalt were truncated to the 99th percentile values and log-transformed (Table S1).

Associations between CERAD and DSST scores and concentrations of metals or their metabolites were assessed individually using multiple linear regression, adjusted for potential demographic and behavioral confounders. The association for each metal and metabolite (log-transformed) with each cognitive function score, adjusted for confounders, was examined. In addition, lead, cadmium, selenium, and methylmercury were all included in one linear regression model to assess their associations with CERAD immediate recall, stratified by age and adjusted for confounders. For descriptive purposes, the average estimated metal concentrations in subjects who performed at the lowest 10th percentile score on CERAD immediate recall (±5%), as well as performance at the 25th, 50th, 75th, and 90th percentile, were determined.

Identified potential confounders, selected based on the literature [15,17,20,32,33], included age (years), sex at birth (male, female), race/ethnicity (Mexican American, Other Hispanic, Non-Hispanic White, Non-Hispanic Black, Non-Hispanic Asian, Other race), education level (high school graduate, more than some college degree), diabetes (yes/no), depression (feel depressed more than 7 days/2weeks, less than 6 days/2weeks), smoking (non-smoker, less than 1 pack/day, more than 1 and less than 2 packs/day, more than 2 packs/day), and alcohol consumption (no-alcohol, 1–4 glasses/day, more than 5 glasses/day).

Models were assessed for overall fit of the data with the F test (*p* < 0.01) and displayed graphically. Leverage that could result in poor estimates of parameters was evaluated using Cook’s D and DFBETAS for the key measures of interest (metals and metabolites). Statistical analyses were performed using R version 4.0.0 (R Core Team, Vienna, Austria) and SAS 9.4 (SAS Institute, Cary, NC, USA).

## 3. Results

### 3.1. Overview of the Study Population

**Figure 1:**
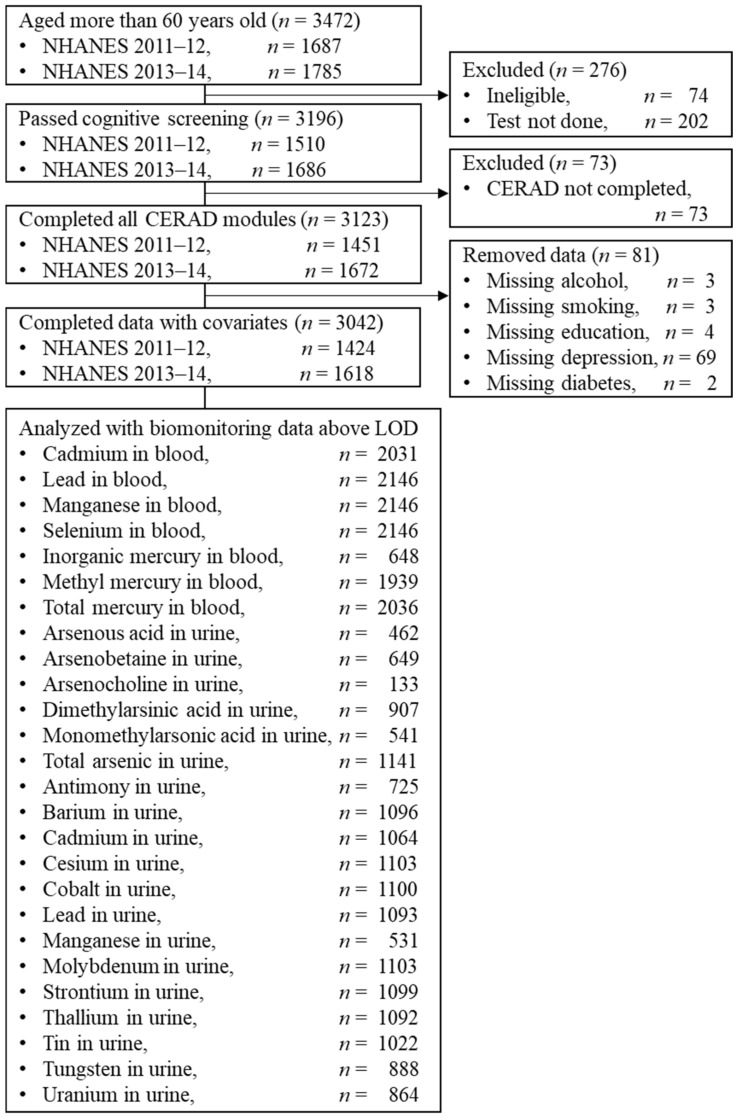
Sampling flow.

A total of 3472 NHANES participants 60 years and older participated in the CERAD modules in the 2011 and 2013 cycles. Of these, 3123 (89.95%) passed the cognitive screening test and completed the CERAD modules, and for 3042 (87.62%), there was sufficient behavioral data for them to be included in the analysis (Figure 1 and Figure S1). The NHANES subsampled some participants for biomonitoring, including the measurement of metals and/or their metabolites in blood or urine. The sample size for each analysis depends on the number of participants with exposure measures (Figure 1 and Table S2).

The distributions of CERAD immediate recall score varied among subgroups of age, sex, diabetes, depression, education level, and alcohol consumption (Table 1). The mean scores were lower in those who were older, male, less educated, and living with diabetes or depression. Individuals with modest alcohol consumption performed better than those with either no or excessive consumption. There was no significant mean difference with cigarette smoking.

### 3.2. Metal Concentrations and Performance on Cognitive Tests

The associations between cognitive function test scores and log-transformed metal concentrations measured in whole blood, adjusted for potential confounders, are shown in Table 2. Cadmium and lead concentrations were significantly associated with decrements in performance on all three cognitive tests. In contrast, higher selenium concentrations were significantly associated with better performance on all cognitive tests. The associations with manganese, total mercury, inorganic mercury, and methylmercury were not statistically significant.

The associations between each cognitive function test score and log-transformed metal concentrations in urine, adjusted for potential confounders, are shown in Table 3. The major metabolites of inorganic arsenic (DMA and MMA) were negatively associated with cognitive function. Arsenic species not metabolized in humans (arsenobetaine, arsenocholine, or organoarsenic) were not associated with cognitive function. Cadmium in urine showed negative associations with CERAD immediate recall and DSST, as it did in blood, and lead showed a negative association with DSST. Tungsten was negatively associated with the scores of CERAD immediate and delayed recall. There were no significant relations found for barium, cobalt, cesium, molybdenum, manganese, strontium, thallium, or uranium.

The associations among four blood metals (cadmium, lead, selenium, and methyl mercury) in one linear regression model on CERAD immediate recall as a function of age are shown in Table 4. While cadmium and lead were negatively associated with CERAD immediate recall scores, selenium showed a strong positive association. All associations were stronger among participants over the age of 70. There was no statistically significant association between blood methylmercury and the CERAD immediate recall score.

The average estimated metal concentrations in subjects who performed at the lowest 10th percentile score on CERAD immediate recall, as well as performance at the 25th, 50th, 75th, and 90th percentiles, are shown in Table 5. For both age groups, those individuals who were in the bottom 10th and 25th percentile of the CERAD immediate recall score had the lowest selenium and the highest lead and cadmium concentrations. Those individuals who scored in the top 75th and 90th percentile groups had the highest selenium and the lowest lead and cadmium concentrations. In general, the analyses found that higher cognitive function levels were associated with higher selenium concentrations and lower levels of lead and cadmium. There was no clear pattern for methylmercury.

## 4. Discussion

### 4.1. Risk Factors from Demographics

The NHANES data show a clear decline in cognitive function with age. Age is a major risk factor for the development of neurodegenerative and ischemic brain diseases, and both genetic factors and environmental exposures can influence the process [34]. There was a clear sex difference. Some studies have shown that while there is no overall difference in intelligence between the sexes, men and women exhibit differences in some cognitive domains [35] and in the risk of developing neurodegenerative diseases [36]. From association analyses, age, sex, and education level were strong covariates, but some metal exposures still affect cognitive function in older adults.

### 4.2. Selenium

The most striking result from this study is the strong positive associations of selenium and cognitive function. Furthermore, better cognitive performance was associated with the combination of higher selenium and lower concentrations of lead and cadmium.

Selenium may counteract the toxic effects of metals through the formation of inert metal–selenium colloids which are then excreted in bile. This was originally described by Hill in 1975, who showed that arsenic, mercury, cadmium, and copper could protect against selenium toxicity [37]. In addition, equimolar complexes are formed between selenium and lead, silver, and zinc [38]. Prior research has found these complexes explain the protection that selenium gives against the toxicity of arsenic and cadmium in the liver, kidney, spleen, brain, or heart [39]. We hypothesize that this creation of inert colloids may at least in part explain our results.

There is some, albeit not very convincing evidence for a beneficial effect of selenium on cognition. Selenium supplementation has not been found to result in significant or beneficial association in clinical studies of dementia. One randomized clinical trial followed 7540 older men over 7 years and found that selenium supplementation did not significantly change dementia incidence [40]. However, a randomized controlled trial of Alzheimer’s disease patients found that super-nutritional selenium supplementation significantly increased selenium concentration in the brain in some patients, and found that those individuals with elevated CSF selenium concentrations did not show the continued decline in the Mini-Mental Examination test performances that was seen in those patients without the elevated brain selenium [41]. Selenium supplementation during pregnancy has been reported to improve the child’s cognitive function [42,43]. In animal experiments, selenium supplementation counterbalanced the adverse effects of lead on hippocampal long-term potentiation and spatial memory [44]. In a case-control study in Taiwan, hearing ability in factory workers was found to be impaired by lead exposure but improved by selenium, which in part counteracted the harmful effect of lead [45]. A positive association between selenium on CERAD scores from the NHANES has been reported [46]. A French study reported that individuals with low serum selenium concentrations did not live as long as those with higher concentrations [47].

Selenium levels often decrease with age [35,48]. Adequate selenium intake from the diet may improve learning and recall abilities by increasing antioxidant activity and by facilitating thyroid action. However, the ability of selenium to counteract the toxicity of other metals is very likely due primarily to the formation of these complexes which are then excreted.

### 4.3. Lead and Cadmium

The harmful associations between lead concentration and cognitive function at all ages is well established. Lead-exposed children in New Zealand, when tested at age 38 years, showed reduced IQ scores in perceptual reasoning and working memory [49]. In Boston, adults aged 45–74 years were found to show a greater decline of story memory and category fluency in relation to tibia lead concentrations [50].

Blood cadmium showed consistent negative associations with immediate learning and recall, delayed recall, and DSST (β range = −2.29, −0.19). Cadmium exposure has negative associations with cognitive ability in children, with sex-specific effects [51]. Prenatal exposure to cadmium, measured in maternal urine during pregnancy, had inverse associations with children’s general cognitive score [52]. These associations were only seen if the mothers were smoking. Tobacco contains volatile cadmium [53]. Children exposed to passive environmental tobacco smoke (ETS), monitored by serum cotinine, have reduced cognitive function [54]. The dose-response shown for ETS is very similar to that for lead, in that there is a steeper decline at low rather than high exposures. It is likely that the decline in cognitive function with ETS is at least in part due to cadmium. Urinary cadmium was inversely associated with a risk of cognitive impairment in older men [55]. The negative cadmium associations with cognition seen in the NHANES data were reported previously [56]. Our results, in conjunction with the previous literature, suggest that exposure to cadmium is negatively associated with cognitive decline in older adults.

### 4.4. Arsenic and Tungsten

MMA and DMA are metabolites of inorganic arsenic, which is the active ingredient in many herbicides [57]. Inorganic arsenic is absorbed in the gastrointestinal tracts from food and drinking water, and is then bio-activated into MMA and DMA [57,58]. The sum of inorganic and methylated arsenic was associated with suppressed fine motor function and processing speed among American Indians aged 64–95 years [59]. Furthermore, arsenic-exposed adults aged 18–60 years showed significantly lower scores on the Mini-Mental State Examination (MMSE) than a control group [60]. There was an inverse dose-dependent relationship between cognitive functions and arsenic exposures [60].

Finding adverse associations for urinary tungsten in the CERAD immediate recall and DSST was unexpected. While tungsten has many uses, it is usually considered to be relatively safe, although it can be detected in soil and potable water [61]. Rats exposed to sodium tungstate have been reported to show subtle neurobehavioral effects [62]. These results suggest that further investigation of the neurobehavioral effects of tungsten in both animals and humans is warranted.

### 4.5. Mercury Compounds

There are three forms of mercury relevant to human health. To our surprise, none of the forms of mercury were associated with CERAD and DSST scores. This is particularly the case for methylmercury, given the evidence from other studies reporting the adverse effects of methylmercury on cognitive performance [20,63]. The methylmercury results may be influenced by fish consumption.

### 4.6. Limitations in Our Study

There are limitations to this study. The study population included adults, 60–80 years of age, living in the community; thus, results cannot be generalized to those living in nursing homes or institutions [29]. Additionally, study participants were required to pass an initial cognitive function screening test before completing the CERAD modules, so the participants had better cognitive function compared to those excluded (<8%). Since we only have included participants with metal concentrations above the LOD, we lost some statistical power. However, this process improves the variance of metal or metabolite concentrations, with the smaller variance under the log-transformed distribution [64]. The range of analyzed participants was 133 to 2146, and any study results are limited by the number of participants. Due to the cross-sectional study design, both metal concentrations and cognitive function were measured only at one point in time; thus, temporal changes in cognitive function due to earlier metal exposure were not assessed. The NHANES data contain only estimated values of omega-3 fatty acids, and these were not included in the model, which could influence methylmercury results. We did not estimate metal concentrations below the LOD. Sensitivity analyses for the key metals, metabolites, and metalloids, using standard estimates (LODs/square root of 2), found minimal change in parameter estimates. We presented unweighted analyses. This may have resulted in small errors in the estimated variances, but has the benefit of treating all participants equally instead of more heavily weighting white respondents. Weighted analyses of the primary models were similar. The model generally fits the data, except for the extreme, highest selenium concentrations, at which point the model slightly overestimates cognitive function. Removing these outliers did not substantially change the parameter estimates.

## 5. Conclusions

We assessed metal exposures and cognitive functions of healthy older adults using the NHANES data. Selenium showed strong positive associations with performance on both CERAD modules and DSST. Concentrations of cadmium, lead, arsenic metabolites, and tungsten, but not mercury compounds, had negative associations with cognitive function. This is consistent with the hypothesis that selenium plays a key role in mitigating the adverse effects of exposure to other toxic metals, primarily through the formation of inert complexes that are then excreted. Because many older adults are selenium deficient, selenium supplementation has the potential to improve cognition. Selenium supplementation prior to the onset of senility warrants further study.

## Figures and Tables

**Table 1:** Mean scores of CERAD immediate recall scores by demographic, behavior, and clinical characteristics ^1^.

Variables	*n* (3042)	CERAD Immediate Recall Mean Scores (95% CI)	*p* Value ^2^
Age categories, years old			
60–69	1615	19.65 (19.42, 19.87)	
≥70	1427	17.47 (17.29, 17.73)	<0.001 ^2^
Sex			
Male	1474	18.02 (17.55, 18.02)	
Female	1568	19.42 (19.17, 19.67)	<0.001 ^2^
Race/Ethnicity			
Non-Hispanic Asian	251	18.49 (17.78, 19.20)	
Mexican American	277	17.73 (17.13, 18.32)	
Other Hispanic	311	17.43 (16.93, 17.92)	
Non-Hispanic Black	733	18.53 (18.18, 18.88)	
Non-Hispanic White	1424	19.16 (18.92, 19.41)	ref
Other race and multi-race	46	17.94 (16.77, 19.10)	<0.001 ^3^
Education			
≤High school graduate	1519	17.43 (17.19, 17.67)	
≥Some college	1523	19.82 (19.59, 20.06)	<0.001 ^2^
Diabetes			
Yes	727	17.89 (17.53, 18.24)	
No	2315	18.86 (18.66, 19.06)	<0.001 ^2^
Depression			
Yes	250	17.29 (16.70, 17.88)	
No	2792	18.74 (18.57, 18.93)	<0.001 ^2^
Smoking per day			
Non-smoker	2661	18.63 (18.46, 18.82)	ref
<1 pack	289	18.47 (17.93, 19.01)	
1–2 pack	84	19.04 (18.06, 20.00)	
≥2 pack	8	18.63 (16.58, 20.70)	0.82 ^3^
Alcohol per day			
No alcohol	1382	17.87 (17.60, 18.13)	ref
1–4 glasses	1564	19.35 (19.12, 19.59)	
≥5 glasses	96	17.76 (16.97, 18.55)	<0.001 ^3^

^1^ ref, reference. ^2^ *p* value was assessed by a *t*-test. ^3^ *p* value was assessed by a Kruskal–Wallis test.

**Table 2:** Linear regression results for whole blood metals (log-transformed) with each cognitive function score, adjusted for sociodemographic, behavior, and clinical characteristics ^1^.

	CERAD Immediate Recall		CERAD Delayed Recall		Digit Symbol Substitution	
	β (95% CI)	*p* Value	β (95% CI)	*p* Value	β (95% CI)	*p* Value
Cadmium, µg/L (*n* = 2031)	−0.54 (−0.90, −0.17)	<0.01	−0.19 (−0.37, −0.01)	0.04	−2.29 (−3.41, −1.16)	<0.01
Lead, µg/L (*n* = 2146)	−0.58 (−0.91, −0.24)	<0.01	−0.19 (−0.35, −0.02)	0.03	−1.08 (−2.12, −0.05)	0.04
Manganese, µg/L (*n* = 2146)	−0.16 (−0.73, 0.42)	0.59	0.24 (−0.05, 0.52)	0.10	0.63 (−1.14, 2.39)	0.48
Selenium, µg/L (*n* = 2146)	2.68 (1.06, 4.30)	<0.01	0.87 (0.06, 1.67)	0.04	8.38 (3.40, 13.36)	<0.01
Total mercury, µg/L (*n* = 2036)	0.20 (−0.03, 0.42)	0.09	0.06 (−0.05, 0.17)	0.29	0.56 (−0.12,1.25)	0.11
Inorganic mercury, µg/L (*n* = 648)	0.58 (−0.31, 1.47)	0.20	−0.33 (−0.76, 0.09)	0.12	−2.35 (−5.07, 0.36)	0.09
Methylmercury, µg/L (*n* = 1939)	0.11 (−0.09, 0.31)	0.28	0.07 (−0.03, 0.17)	0.17	0.21 (−0.40, 0.83)	0.50

^1^ Models adjusted for age, sex, race/ethnicity, education level, depression, diabetes, cigarette smoking, and alcohol consumption.

**Table 3:** Linear regression results for urinary metals (log-transformed) in association with each cognitive function score, adjusted for sociodemographic, behavior, and clinical characteristics ^1^.

	CERAD Immediate Recall		CERAD Delayed Recall		Digit Symbol Substitution	
	β (95% CI)	*p* Value	β (95% CI)	*p* Value	β (95% CI)	*p* Value
Total Arsenic, µg/L (*n* = 1141)	0.02 (−0.22, 0.64)	0.86	−0.06 (−0.18, 0.06)	0.35	0.27 (−0.47, 1.00)	0.48
Arsenous Acid, µg/L (*n* = 462)	−0.42 (−1.20, 0.37)	0.30	−0.10 (−0.49, 0.29)	0.61	2.19 (−0.29, 4.68)	0.08
Arsenobetaine, µg/L (*n* = 649)	0.09 (−0.20, 0.37)	0.56	−0.02 (−0.16, 0.12)	0.83	0.84 (−0.03, 1.72)	0.06
Arsenocholine, µg/L (*n* = 133)	0.37 (−0.57, 1.30)	0.44	0.07 (−0.35, 0.50)	0.74	−0.02 (−2.69, 2.65)	0.99
Dimethylarsinic Acid, µg/L (*n* = 907)	−0.20 (−0.65, 0.25)	0.39	−0.23 (−0.46, −0.01)	0.04	−1.31 (−2.69, 0.07)	0.06
Monomethylarsonic Acid, µg/L (*n* = 541)	−0.90 (−1.48, −0.33)	<0.01	−0.37 (−0.66, −0.08)	0.01	−0.48 (−2.21, 1.25)	0.59
Barium, µg/L (*n* = 1096)	−0.06 (−0.34, 0.22)	0.68	−0.03 (−0.17, 0.11)	0.72	0.50 (−0.36, 1.36)	0.25
Cadmium, µg/L (*n* = 1064)	−0.31 (−0.63, 0.001)	0.05	−0.06 (−0.22, 0.10)	0.46	−1.42 (−2.38, −0.46)	<0.01
Cobalt, µg/L (*n* = 1100)	0.11 (−0.23, 0.45)	0.52	0.06 (−0.11, 0.22)	0.52	0.26 (−0.76, 1.27)	0.62
Cesium, µg/L (*n* = 1103)	0.05 (−0.36, 0.46)	0.82	−0.005 (−0.21, 0.20)	0.97	−0.27 (−1.52, 1.00)	0.68
Molybdenum, µg/L (*n* = 1103)	−0.13 (−0.44, 0.18)	0.41	−0.06 (−0.21, 0.10)	0.47	−0.06 (−1.01, 0.88)	0.89
Manganese, µg/L (*n* = 531)	0.06 (−0.64, 0.77)	0.86	−0.01 (−0.37, 0.35)	0.96	0.35 (−1.84, 2.55)	0.75
Lead, µg/dL (*n* = 1093)	−0.26 (−0.58, 0.06)	0.12	−0.03 (−0.19, 0.13)	0.71	−1.03 (−2.01, −0.06)	0.04
Antimony, µg/L (*n* = 725)	−0.56 (−1.13, 0.01)	0.06	−0.27 (−0.56, 0.03)	0.08	−1.30 (−3.10, 0.50)	0.16
Tin, µg/L (*n* = 1022)	−0.16 (−0.41, 0.08)	0.20	−0.08 (−0.20, 0.05)	0.22	−0.68 (−1.44, 0.08)	0.08
Strontium, µg/L (*n* = 1099)	0.05 (−0.32, 0.41)	0.81	0.08 (−0.10, 0.26)	0.39	0.70 (−0.42, 1.82)	0.22
Thallium, µg/L (*n* = 1092)	0.12 (−0.30, 0.54)	0.58	0.09 (−0.12, 0.30)	0.38	0.49 (−0.79, 1.77)	0.45
Tungsten, µg/L (*n* = 888)	−0.38 (−0.75, −0.01)	0.04	−0.19 (−0.38, −0.004)	0.05	−0.47 (−1.62, 0.68)	0.42
Uranium, µg/L (*n* = 864)	−0.11 (−0.49, 0.26)	0.55	−0.12 (−0.31, 0.07)	0.22	0.34 (−1.11, 1.45)	0.55

^1^ Models adjusted for age, sex, race/ethnicity, education levels, depression, diabetes, cigarette smoking, and alcohol consumption.

**Table 4:** Linear regression results for multiple blood metals (log-transformed) on CERAD immediate recall as a function of age, adjusted for sociodemographic, behavior, and clinical characteristics, stratified by age group into 60s and more than 70 years old ^1^.

	Stratified 60s Years Old Group (*n* = 984)		Stratified ≥ 70s Years Old Group (*n* = 851)	
	β (95% CI)	*p* Value	β (95% CI)	*p* Value
Lead, µg/L	−0.37 (−0.87, 0.13)	0.14	−0.85 (−1.44, −0.27)	<0.01
Cadmium, µg/L	−0.53 (−1.05, −0.01)	0.05	−0.68 (−1.32, −0.04)	0.04
Selenium, µg/L	2.62 (0.22, 5.03)	0.03	3.44 (0.68, 6.21)	0.01
Methylmercury, µg/L	0.03 (−0.24, 0.31)	0.80	0.16 (−0.17, 0.48)	0.34

^1^ Models adjusted for sex, race/ethnicity, education levels, depression, diabetes, cigarette smoking, and alcohol consumption.

**Table 5:** Average values of metal concentrations of selected percentage of CERAD immediate recall scores (±5%) ^1^.

Percentage of CERAD Immediate Recall Score: Estimated Value Based on Model	Estimated CERAD Immediate Recall Score	Selenium (µg/L)	Lead (µg/L)	Cadmium (µg/L)	Methylmercury (µg/L)
Aged 60–69 years old, (*n* = 984)					
10%	17.03	190.31	29.72	0.60	1.91
25%	18.09	190.44	18.90	0.69	1.69
50%	19.54	198.67	17.38	0.58	2.06
75%	20.88	206.39	15.18	0.49	2.28
90%	22.00	191.83	15.54	0.51	2.10
Aged 70–89 years old, (*n* = 851)					
10%	14.69	184.47	25.22	0.65	1.28
25%	15.98	190.75	23.57	0.65	2.17
50%	17.46	196.98	17.83	0.49	1.77
75%	18.83	200.66	16.96	0.49	2.10
90%	19.80	198.97	15.30	0.51	1.86

^1^ Models included log-transformations of metal concentrations to fit the data. Analyses were adjusted for sex, race/ethnicity, education level, depression, diabetes, cigarette smoking, and alcohol consumption. All parameters and their *p*-values are provided in Table 4. Percentile data represent average estimated CERAD immediate recall score at each percentile listed (±5%) to provide stable estimates. Metal concentrations are average estimated values for persons with CERAD immediate recall scores.

## Data Availability

Publicly available datasets were analyzed in this study. The data can be found at https://wwwn.cdc.gov/nchs/nhanes/Default.aspx (accessed on 15 October 2019).

## Supplementary Materials

The following supporting information can be downloaded at: https://www.mdpi.com/article/10.3390/ijerph19042327/s1, Figure S1: Distribution of CERAD immediate recall, delayed recall, and DSST modules; Table S1: Distribution of metal concentrations that were analyzed with multivariable linear regression; Table S2: Number of cases below LOD and missing samples in measured metals.
