# Supplementary material for: Associations between Metal Exposures and Cognitive Function in American Older Adults
Source: Int J Environ Res Public Health. 2022 Feb 17;19(4):2327. doi: 10.3390/ijerph19042327 (PMC8871766; doi:10.3390/ijerph19042327)

“Online Supplementary Material” Associations of metal exposures and cognitive function in American older adults, N. Sasaki

SUPPLEMENTAL TABLES

Table S1. Distribution of metal concentrations which analyzed with multivariable linear regression

	**Minimum**	**25 percentiles**	**Median**	**Mean**	**75 percentiles**	**95 percentiles**	**99 percentiles**	**Maximum**
Cognitive function modules								
CERAD immediate recall, (*n* = 3,042)	0	16	19	18.63	22	26	28	30
CERAD delayed, (*n* = 3,042)	0	4	6	5.81	8	9	9	10
DSST, (*n* = 2,904)	0	33	46	45.69	58	74	84	105
Metals in blood								
Cadmium, µg/L, (*n* = 2,031)	0.10	0.27	0.40	0.55	0.65	1.48	2.50	3.60
Lead, µg/L, (*n* = 2,146)	2.6	10.10	14.70	19.00	22.20	43.90	84.80	270.0
Manganese, µg/L (*n* = 2,146)	1.75	6.96	8.74	9.41	11.17	15.52	20.81	62.51
Selenium, µg/L (n = 2,146)	105.39	177.02	192.45	194.64	208.22	237.27	278.38	734.80
Total mercury, µg/L (*n* =2,036)	0.16	0.56	1.02	1.83	2.03	6.04	12.88	36.99
Inorganic mercury, µg/L (*n* = 648)	0.27	0.32	0.41	0.50	0.58	1.00	1.90	3.70
Methylmercury, µg/L (*n* = 1,939)	0.12	0.41	0.87	1.74	1.96	6.05	12.93	38.55
Metals in urine								
Total Arsenic, µg/L (*n* = 1,141)	0.45	3.78	7.93	18.87	18.25	59.12	163.02	1269.00
Arsenous Acid, µg/L (*n* = 462)	0.12	0.49	0.63	0.82	0.82	1.37	2.14	66.30
Arsenobetaine, µg/L (*n* = 649)	1.16	2.66	6.47	18.95	16.60	70.80	186.08	1140.00
Arsenocholine, µg/L (*n* = 133)	0.11	0.21	0.35	0.77	0.60	2.55	6.37	19.23
DMA, µg/L (*n* = 907)	1.80	3.01	4.53	6.95	7.66	18.30	39.20	136.00
MMA, µg/L (*n* = 541)	0.20	0.49	0.83	1.14	1.23	2.39	3.70	72.90
Barium, µg/L (*n* = 1,096)	0.06	0.46	0.89	1.62	1.82	4.54	8.16	87.40
Cadmium, µg/L (*n* = 1,064)	0.04	0.19	0.35	0.52	0.64	1.62	2.71	4.89
Cobalt, µg/L (*n* = 1,100)	0.04	0.20	0.32	0.57	0.51	1.34	4.84	33.72
Caesium, µg/L (*n* = 1,103)	0.28	2.62	4.16	4.78	6.13	10.50	16.80	29.70
Molybdenum, µg/L (*n* = 1,103)	1.78	19.70	35.73	47.62	62.00	129.00	221.19	442.00
Manganese, µg/L (*n* = 531)	0.08	0.13	0.17	0.29	0.24	0.45	2.17	18.95
Lead, µg/dL (*n* = 1,093)	0.05	0.27	0.46	0.72	0.81	1.99	4.80	13.40
Antimony, µg/L (*n* = 725)	0.02	0.04	0.06	0.09	0.09	0.21	0.69	4.23
Tin, µg/L (*n* = 1,022)	0.09	0.40	0.75	2.02	1.70	6.33	29.24	71.00
Strontium, µg/L (*n* = 1,099)	2.83	45.65	79.42	118.76	130.95	268.59	554.20	7565.49
Thallium, µg/L (*n* = 1,092)	0.02	0.08	0.14	0.16	0.21	0.38	0.60	1.21
Tungsten, µg/L (*n* = 888)	0.02	0.04	0.07	0.15	0.12	0.37	1.56	17.61
Uranium, µg/L (*n* = 864)	0.002	0.005	0.008	0.02	0.014	0.044	0.145	0.722

Table S2. Number of below LOD and missing samples in measured metals[1]

	LOD value in 2011 - 2012	LOD value in 2013 - 2014	Used in model, *n*[1]	Above LOD, *n*	Below LOD, *n*	Missing, *n*	Subgroup, *n*
Metals in blood							
Cadmium, µg/L	0.16	0.10	2,031	2,071	116	0	2,187
Lead, ng/dL	0.25	0.07	2,146	2,187	0	0	2,187
Manganese, µg/L	1.06	0.99	2,146	2,187	0	0	2,187
Selenium, µg/L	30.00	24.28	2,146	2,187	0	0	2,187
Total mercury, µg/L	0.16	0.28	2,036	2,070	117	0	2,187
Inorganic mercury, µg/L	0.27	0.27	648	660	1521	0	2,181
Methylmercury, µg/L	0.12	0.12	1,939	1,973	208	0	2,181
Metals in urine							
Total Arsenic, µg/L	1.25	0.26	1,141	1,143	19	11	1,173
Arsenous Acid, µg/L	0.48	0.12	462	462	711	0	1,173
Arsenobetaine, µg/L	1.19	1.16	649	650	523	0	1,173
Arsenocholine, µg/L	0.28	0.11	133	134	1039	0	1,173
DMA, µg/L	1.80	1.91	907	908	265	0	1,173
MMA, µg/L	0.89	0.20	541	542	631	0	1,173
Barium, µg/L	0.10	0.06	1,096	1,096	7	27	1,130
Cadmium, µg/L	0.06	0.04	1,064	1,064	39	27	1,130
Cobalt, µg/L	0.05	0.02	1,100	1,100	3	27	1,130
Cesium, µg/L	0.12	0.09	1,103	1,103	0	27	1,130
Molybdenum, µg/L	0.99	0.80	1,103	1,103	0	27	1,130
Manganese, µg/L	0.08	0.13	531	531	572	27	1,130
Lead, µg/dL	0.08	0.03	1,093	1,093	10	27	1,130
Antimony, µg/L	0.04	0.02	725	725	378	27	1,130
Tin, µg/L	0.22	0.09	1,022	1,022	81	27	1,130
Strontium, µg/L	2.50	2.34	1,099	1,099	1	30	1,130
Thallium, µg/L	0.02	0.02	1,092	1,092	11	27	1,130
Tungsten, µg/L	0.03	0.02	888	888	211	31	1,130
Uranium, µg/L	0.003	0.002	864	864	239	27	1,130

[1]When participants are not recorded complete demographic data, they were not included in statistical analysis. Therefore, study population is not the same number with number of above LOD.

“Online Supplementary Material” Associations of metal exposures and cognitive function in American older adults, N. Sasaki

SUPPLEMENTAL FIGURE

Figure S1. Distribution of CERAD immediate recall, delayed recall and DSST modules.

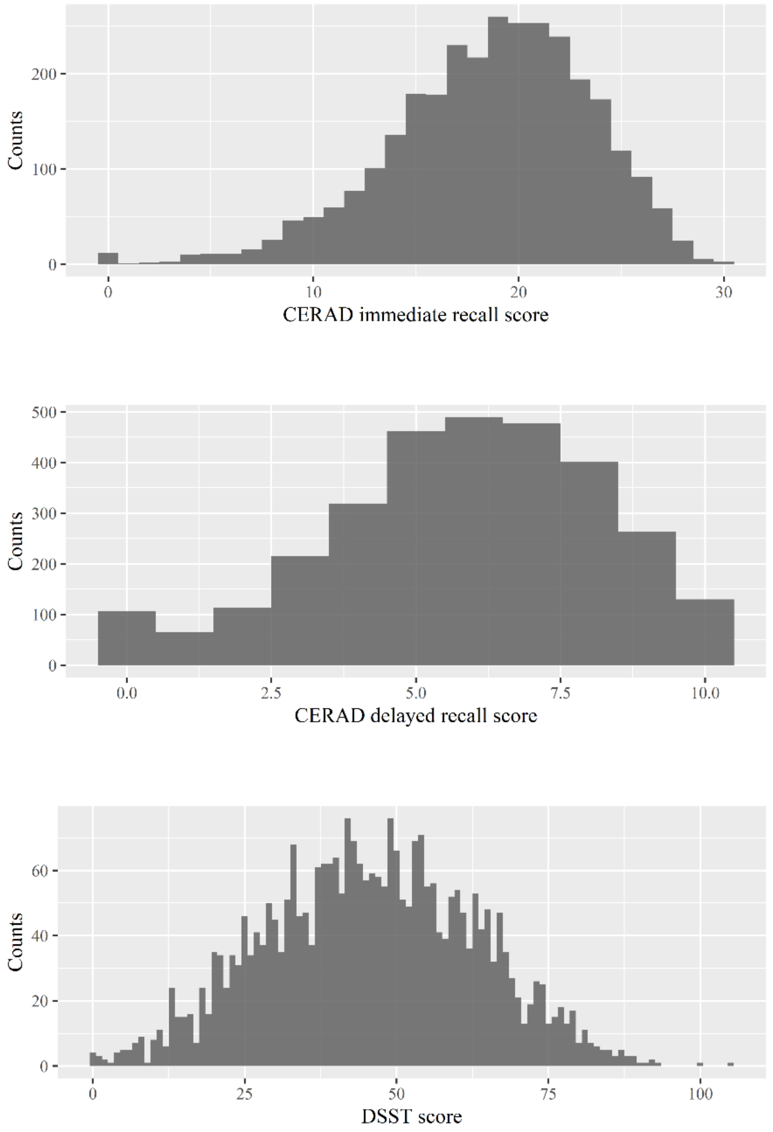

Supplement: Supplementary file 1 [file ijerph-19-02327-s001.zip › ijerph-1582585-supplementary.pdf]
